# A Novel Hybrid of a Fading Filter and an Extreme Learning Machine for GPS/INS during GPS Outages

**DOI:** 10.3390/s18113863

**Published:** 2018-11-10

**Authors:** Di Wang, Xiaosu Xu, Yongyun Zhu

**Affiliations:** 1 Key Laboratory of Micro-Inertial Instrument and Advanced Navigation Technology, Ministry of Education, Southeast University, Nanjing 210096, China; 230189694@seu.edu.cn (D.W.); zhyy@seu.edu.cn (Y.Z.); 2 School of Instrument Science and Engineering, Southeast University, Nanjing 210096, China

**Keywords:** fading filter, extreme learning machine, GPS/INS, integrated navigation

## Abstract

In this paper, a novel algorithm based on the combination of a fading filter (FF) and an extreme learning machine (ELM) is presented for Global Positioning System/Inertial Navigation System (GPS/INS) integrated navigation systems. In order to increase the filtering accuracy of the model, a variable fading factor fading filter based on the fading factor is proposed. It adjusts the fading factor by the ratio of the estimated covariance before and after the moment which proves to have excellent performance in our experiment. An extreme learning machine based on a Fourier orthogonal basis function is introduced that considers the deterioration of the accuracy of the navigation system during GPS outages and has a higher positioning accuracy and faster learning speed than the typical neural network learning algorithm. In the end, a simulation and real road test are performed to verify the effectiveness of this algorithm. The results show that the accuracy of the fading filter based on a variable fading factor is clearly improved, and the proposed improved ELM algorithm can provide position corrections during GPS outages more effectively than the other algorithms (ELM and the traditional radial basis function neural network).

## 1. Introduction

With the rapid development of the intelligent transportation system (ITS), vehicle navigation and positioning has attracted more and more researchers in recent years. In most systems, a combination of the Global Positioning System (GPS) and the Inertial Navigation System (INS) is used as the main positioning system. GPS receivers can provide high-precision navigation and positioning information by tracking at least four satellites. However, the performance of standalone GPS receivers may deteriorate under conditions such as GPS signal outages due to multipath effects [1,2]. However, INS is a self-contained system with excellent concealment, which makes it free from external environment interference. It obtains the position and velocity of a vehicle using the inertial measurement unit (IMU), which consists of three-axis accelerometers and three-axis rate gyros [3]. Unfortunately, errors in INS may increase over time because of noise, bias instability errors, dependent random noise, and random-work errors. Therefore, GPS is usually integrated with INS to restrain the accumulated positioning errors [4]. Currently, GPS/INS is considered to be the best style of vehicle navigation system.

GPS and INS data are processed synchronously by the fusion algorithm, which plays an important role in the accuracy of GPS/INS integrated navigation systems. As a linear optimal estimation algorithm, the Kalman filter (KF) is known as the most popular algorithm for GPS/INS integrated navigation at present. Scholars have conducted many studies based on KF to enhance the performance of combined navigation systems. So, an extended Kalman filter (EKF) algorithm was introduced in reference [5] to overcome the deficiencies of KF, which cannot handle non-linear data. EKF truncates the first-order linearization of Taylor’s expansion of the nonlinear function and ignores the remaining higher-order terms, thereby transforming the nonlinear problem into a linearity. Therefore, the estimation error caused by the linearization is reduced. References [6,7] introduced an unsupervised Kalman filter (UKF) into an integrated navigation system. Based on the unscented transform (UT), the UKF uses UT to estimate the mean and covariance, the accuracy of which can reach the fourth-order of the Taylor series, so it is better than EKF. Due to the volatile working environment of the integrated navigation system, the parameters of the navigation model as well as the statistical characteristics of the noise may also change from time to time. An adaptive Kalman filter that can correct system errors in real-time was proposed to allow adaptation to environmental changes [8]. Many studies have been based on the adaptive Kalman filter, such as the information adaptive estimation Kalman filter (IAE-KF) and the multi-model adaptive estimation Kalman filter (MMAE-KF), as well as the robust adaptive cubature Kalman filter (RACKF) [9,10,11]. Although the conventional algorithm-based Kalman filter has greatly improved the accuracy of the GPS/INS integrated navigation system, it does not suppress error divergence when the GPS signal is missing. Therefore, it is necessary to find a better way to solve the problem of combined GPS/INS systems during GPS outages in harsh environments.

In recent years, with the rapid development of artificial intelligence (AI) technology, a lot of scholars began to apply AI-aided KF to GPS/INS integration in order to reduce the GPS signal loss. The most common method is to estimate the error by AI instead of the Kalman filter to correct the inertial navigation system when the GPS signal is interrupted. Some AI algorithms have been widely used to improve the performance of the system, for instance, fuzzy logic and neural networks. An adaptive fuzzy logic method was introduced in reference [12] which suppressed the divergence of inertial navigation errors by establishing an INS error model. Reference [13] proposed a combined navigation method based on the back propagation neural network (BPNN), which trains the model parameters when GPS is available, predicts the output of the Kalman filter, and corrects the result of the INS solution when GPS is unavailable. However, it has difficulty meeting the highly dynamic requirements of the navigation system due to its slow training speed and poor real-time performance. In references [14,15,16], the radical basis function neural network (RBFNN) was used to improve the accuracy of the navigation system during GPS outages. The RBFNN was shown to have a faster training speed and better real-time performance than BPNN.

More algorithms with faster training and more generalization ability have been proposed following the massive amount of research on AI algorithms. An extreme learning machine (ELM) algorithm was proposed by Professor Huang Guangbin in 2004 [17]. In contrast to the traditional neural network, ELM is a single-layer feed-forward neural network in which all the hidden layer parameters are randomly generated and do not require a tedious iterative process. There are many advantages of ELM, such as fast training, good classification results, and so on. Therefore, it is widely used in the fields of model prediction, fault diagnosis, speech recognition, and image recognition [18,19,20]. Li et al. [21] presented a method based on map matching and an extreme learning machine for taxi GPS data, which has superior performance in matching accuracy. Reference [22] presented an extreme learning machine as a mechanism for learning stored digital elevation information to aid unmanned aerial vehicles in navigation through terrain.

To achieve good performance for GPS/INS during GPS outages, an extreme learning machine is introduced and improved in this paper. The improved extreme learning machine based on a Fourier orthogonal basis function, named IELM, is proposed. This algorithm has the advantages of fast training, satisfying the real-time requirements of the navigation system, and good generalization performance, which can improve the convergence performance of network training of conventional ELM. This algorithm can slow down the process of error accumulation in neural network prediction, maintain the stability of the system, and restrain the divergence of navigation information when the GPS signal is out of lock. In order to further improve the navigation accuracy of GPS/INS, an improved fading filter (IFF) is proposed in this paper. The improved fading filter algorithm adaptively changes the fading factor in real-time according to the covariance in a fading filter (FF), which effectively overcomes the problem of filter divergence.

The rest of this paper is organized as follows: in Section 2, the GPS/INS integrated navigation error models are introduced, such as the equation of state, the speed error equation, the position error equation, and so on. In Section 3, the proposed algorithms—the improved fading filter and the improved extreme learning machine—are described. A novel fusion algorithm for GPS/INS systems is introduced in Section 4. In Section 5, the performance of the proposed algorithm is verified by simulation experiments and vehicle tests. The conclusions are given in Section 6.

## 2. The Position from the GPS/INS System

In this section, the error model of INS is listed and the loosely coupled GPS/INS integrated navigation system with a 15-state vector is discussed in detail.

### 2.1. Error Modeling

Select the “East, North, Up (ENU)” geographical coordinate system (g) as the inertial navigation system navigation reference frame, remembered as the n department. The n department, as the reference frame of the differential equation of attitude, can be expressed as

(1) $${\dot{C}}_{b}^{n}=C_{b}^{n}\left(\omega_{nb}^{b}\times \right)$$

where b denotes the body frame; $C_{b}^{n}$ denotes the attitude matrix or direction cosine matrix, which can be used to transform from frame b to frame n; $\omega_{nb}^{b}$ denotes the angular rate vector of frame b with respect to the navigation frame n; (⋅×) denotes the skew-symmetric matrix. Since the gyroscope outputs the angular rate vector of frame b to the inertial frame n, the velocity information $\omega_{nb}^{b}$ cannot be directly measured. So, Equation (1) needs to be transformed, as follows [23]:

(2) $${\dot{C}}_{b}^{n}=C_{b}^{n}\left(\omega_{ib}^{b}\times \right)-\left(\omega_{in}^{n}\times \right)C_{b}^{n}$$

where $\omega_{ib}^{b}$ denotes the output of the gyroscope, and $\omega_{in}^{n}$ is the rotation of frame n to inertial frame i, which can be expressed as

(3) $$\omega_{in}^{n}=\omega_{ie}^{n}+\omega_{en}^{n}$$

(4) $$\left\{ \begin{array}{l} \omega_{ie}^{n}={\left[\begin{array}{ccc} 0 & \omega_{ie}\cos L & \omega_{ie}\sin L \end{array}\right]}^{T}\\ \omega_{en}^{n}={\left[\begin{array}{ccc} -\frac{V_{N}}{R_{M}+h} & \frac{V_{E}}{R_{N}+h} & \frac{V_{E}}{R_{N}+h}\tan L \end{array}\right]}^{T} \end{array}\right.$$

where $\omega_{ie}^{n}$ denotes the earth self-rotation rate in frame n; $\omega_{en}^{n}$ denotes the angle rate of frame n relative to frame e in frame n; e denotes the earth frame; L and h denote the local latitude and altitude, respectively; $V_{N}$ and $V_{E}$ denote the north and east speeds of the vehicle, respectively; $R_{M}$ denotes the radius of curvature in the meridian; and $R_{N}$ denotes the radius of the curvature the prime vertical direction.

The specific force equation of INS is another basic equation, which can be expressed as

(5) $${\dot{V}}_{en}^{n}=C_{b}^{n}f_{sf}^{b}-\left(2\omega_{ie}^{n}+\omega_{en}^{n}\right)\times V_{en}^{n}+g^{n}$$

where $f_{sf}^{b}$ is the vehicle ground specific force measured by the accelerometer; $V_{en}^{n}$ is the vehicle velocity in frame n; and $g^{n}$ is the gravitational acceleration.

Last, the position information of INS can be given follows:

(6) $$\left\{ \begin{array}{l} \dot{\lambda }=\frac{\sec L}{R_{N}+h}V_{E}^{n}\\ \dot{L}=\frac{1}{R_{M}+h}V_{N}^{n}\\ \dot{h}=V_{U}^{n} \end{array}\right.$$

where:

(7) $$R_{M}=\frac{R_{N}\left(1-d^{2}\right)}{1-d^{2}\sin^{2}L},\;R_{N}=\frac{R_{e}}{{\left(1-d^{2}\sin^{2}L\right)}^{1/2}},\;d=\sqrt{2f-f^{2}}$$

where λ is the local longitude, which is provided by GPS; d is the Earth’s elliptical eccentricity; and f is the flattening of the earth.

By ignoring the effects of some small quantities, the linear and simple error equations of INS can be expressed as

(8) $$\dot{\phi }=\phi \times \omega_{in}^{n}+\delta \omega_{in}^{n}-\delta \omega_{ib}^{n}$$

(9) $$\delta {\dot{V}}^{n}=f_{sf}^{n}\times \phi +V^{n}\times \left(2\delta \omega_{ie}^{n}+\delta \omega_{en}^{n}\right)\times \delta V^{n}+\delta f_{sf}^{n}+\delta g^{n}$$

(10) $$\delta \dot{L}=\frac{1}{R_{M}+h}\delta V_{N}-\frac{V_{N}}{{\left(R_{M}+h\right)}^{2}}\delta h$$

(11) $$\delta \dot{\lambda }=\frac{\sec L}{R_{N}+h}\delta V_{E}+\frac{V_{E}\sec L\tan L}{R_{N}+h}\delta L-\frac{V_{E}\sec L}{{\left(R_{N}+h\right)}^{2}}\delta h$$

(12) $$\delta \dot{h}=\delta V_{U}$$

where Equation (8) denotes the attitude error equation; Equation (9) denotes the speed error equation; and Equations (10)–(12) denote the position error equation.

### 2.2. The Model of the GPS/INS Loosely Coupled Integrated System

**Figure 1:**
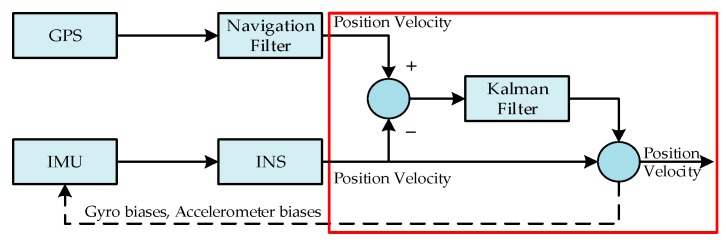
Architecture of the loosely coupled Global Positioning System (GPS)/Inertial Navigation System (INS) integrated system.

The navigation system based on GPS/INS can overcome the shortcomings of each navigation device and enhance the performance of the overall system. At present, there are two commonly used navigation methods: loosely coupled and tightly coupled. Considering that the loosely coupled method is easy to implement and the calculation process is simple [24], this method was chosen as the research object in this paper. The architecture of the loosely coupled GPS/INS integrated system is shown as Figure 1. The velocity difference and position difference, which are calculated by the information between GPS and INS, are the KF inputs. The INS navigation information is corrected by the output information of KF, which includes the velocity difference, position difference, gyro biases, and accelerometer biases.

According to the loosely coupled integrated system based on the Kalman filter, the state equation and measurement equation can be expressed as

(13) $$\left\{ \begin{array}{l} \dot{X}=FX+GW\\ Z=HX+V \end{array}\right.$$

where F represents the state transition matrix; G represents the state noise matrix; W represents the process noise vector; Z represents the measurement vector; H represents the measurement matrix; and V represents the measurement noise matrix. A 15-state vector was proposed for the experiment in this paper. The state vector X is given by

(14) $$X={\left[\begin{array}{ccccccccccccccc} \phi_{E} & \phi_{N} & \phi_{U} & \delta V_{E} & \delta V_{N} & \delta V_{U} & \delta L & \delta \lambda & \delta h & \nabla_{x} & \nabla_{y} & \nabla_{z} & \varepsilon_{x} & \varepsilon_{y} & \varepsilon_{z} \end{array}\right]}^{T}.$$

The details of the state vectors are listed in Table 1.

A 6-dimension measurement vector was designed in the loosely coupled GPS/INS integrated system in this study, which includes a 3-dimension speed error and 3-dimension position error between INS and GPS. The measurement vector Z can be expressed as

(15) $$Z=\left[\begin{array}{c} V_{INS}^{n}-V_{GPS}^{n}\\ P_{INS}-P_{GPS} \end{array}\right]$$

where $V_{INS}^{n}$ and $P_{INS}$ denote the speed and location of the inertial navigation system, and $V_{GPS}^{n}$ and $P_{GPS}$ denote the speed and location information provided by GPS.

## 3. Proposed Algorithms

In this section, the principle of the fading filter is described, and a new, improved fading factor algorithm is proposed. Then, an ELM algorithm based on a single hidden layer feed-forward neural network is discussed, while an improved ELM algorithm with a Fourier orthogonal basis function is introduced.

### 3.1. Principles of the Fading Filter

All of the historical measurements are utilized comprehensively in the standard Kalman filter, and the optimal estimation of the state can be obtained theoretically when the filtering model is accurate. After long-time filtering, the filtering gain calculation loop generally converges gradually and the filtering gain decreases, which makes the inertia of the filter increase, and the correction effect of the new measurement value on the state estimation decreases gradually. In order to overcome this problem, researchers proposed a fading filter algorithm to modify the system noise and the weight of measurement noise in the filtering process to gradually reduce the weight of the historical information and achieve the purpose of reducing the filter inertia. A fading filter improves the filtering accuracy under the condition of inaccurate system modeling; it is a sub-optimal filtering algorithm.

The loosely coupled integrated system model in Equation (13) is transformed into the discrete time formula:

(16) $$\left\{ \begin{array}{l} X_{k}=\Phi_{k,k-1}X_{k-1}+G_{k}W_{k}\\ Z_{k}=H_{k}X_{k}+V_{k} \end{array}\right..$$

The time update is

(17) $${\hat{X}}_{k,k-1}=\Phi_{k,k-1}{\hat{X}}_{k-1}$$

(18) $$P_{k,k-1}=S_{k}\Phi_{k,k-1}P_{k-1}\Phi_{k,k-1}^{T}+G_{k,k-1}Q_{k-1}G_{k,k-1}^{T}$$

where ${\hat{X}}_{k,k-1}$ denotes the predicted state estimate; $\Phi_{k,k-1}$ denotes the state transition matrix; $P_{k,k-1}$ denotes the predicted estimate covariance; $S_{k}$ denotes the fading factor; and $S_{k}\geq 1$.

The measurement update is

(19) $$K_{k}=P_{k,k-1}H_{k}^{T}{\left[H_{k}P_{k,k-1}H_{k}^{T}+R_{k}\right]}^{-1}$$

(20) $$\left\{ \begin{array}{l} {\hat{X}}_{k}={\hat{X}}_{k,k-1}+K_{k}\left[Z_{k}-H_{k}{\hat{X}}_{k,k-1}\right]\\ P_{k}=\left[I-K_{k}H_{k}\right]P_{k-1} \end{array}\right.$$

where $K_{k}$ represents the Kalman filter gain, and $Q_{k}$ and $R_{k}$ represent the covariance matrices of the state noise and measurement noise, respectively, which can be calculated as

(21) $$Q_{k}=E\left[W_{k}W_{k}^{T}\right],\;R_{k}=E\left[V_{k}V_{k}^{T}\right].$$

All of the above processes make up the fading filter. It can be regarded as a traditional Kalman filter when $S_{k}=1$ is satisfied in the fading filter. If $S_{k}$ is greater than 1, the historical information is forgotten faster in the fading filter [25].

### 3.2. The Improved Fading Factors

The choice of fading factor plays a key role in the performance of the fading filter. The fading factor of conventional fading filtering algorithms is usually chosen empirically. In reference [26], a recursive least squares (RLS) based variable fading factor algorithm was proposed. Reference [27] introduced a multiple fading factor calculation method. It is very difficult to apply these methods to the GPS/INS system because of the complex computation required. So, a simplified and well-behaved algorithm is proposed in this section.

The covariance matrix of the measurement prediction error $\sigma_{k}^{2}$ is defined as

(22) $$\sigma_{k}^{2}=E\left[\left(Z_{k}-H_{k}{\hat{X}}_{k,k-1}\right){\left(Z_{k}-H_{k}{\hat{X}}_{k,k-1}\right)}^{T}\right].$$

When $\sigma_{k}^{2}$ increases, it satisfies $\sigma_{k}^{2}-\sigma_{k-1}^{2}>0$; at the same time, the filter is in a divergent state. We should increase the Kalman filter gain and the system noise variance matrix to emphasize the effect of the new data on the filtering—the larger the $\sigma_{k}^{2}$ value is, the greater the correction must be. According to the above description, an algorithm for calculating variable fading factor is proposed:

(23) $$S_{k}=S_{k-1}+u\cdot \beta$$

(24) $$P_{k,k-1}=\Phi_{k,k-1}P_{k-1}\Phi_{k,k-1}^{T}+\frac{1}{S_{k}}G_{k,k-1}Q_{k-1}G_{k,k-1}^{T}$$

(25) $$K_{k}=\frac{1}{S_{k}}P_{k,k-1}H_{k}^{T}{\left[H_{k}P_{k,k-1}H_{k}^{T}+R_{k}\right]}^{-1}$$

where u represents the filter step, and β represents the ratio of estimated covariance before and after the moment. In the filtering process, β=−1 is taken if the covariance of the estimated error $\sigma_{k}^{2}$ tends to increase and the ratio of the previous two times exceeds the limits, otherwise, β=1.

(26) $$\beta =\{\begin{array}{cc} 1 & ,\frac{\sigma_{k}^{2}}{\sigma_{k-1}^{2}}\geq m\\ -1 & ,\frac{\sigma_{k}^{2}}{\sigma_{k-1}^{2}}<m \end{array}.$$

On the other hand, the value of $S_{k}$ needs to be corrected in order to prevent the filter from diverging out of the limited range.

(27) $$S_{k}=\{\begin{array}{cc} 1 & ,S_{k}>1\\ S_{\min} & ,S_{k}<S_{\min}\\ S_{k} & ,other \end{array}.$$

**Figure 2:**
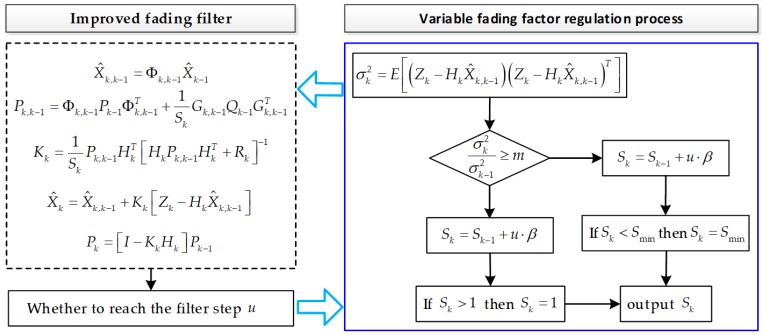
Flow chart of the proposed improved fading filter algorithm.

In summary, in the filtering process, if $\sigma_{k}^{2}$ is not high, then $S_{k}$ will eventually approach 1, and the filter will be in a steady state. If there is a large deviation $\sigma_{k}^{2}$, $S_{k}$ is reduced to achieve the purpose of highlighting the new data correction. Figure 2 shows the proposed improved fading filtering algorithm (IFF).

### 3.3. Extreme Learning Machine

**Figure 3:**
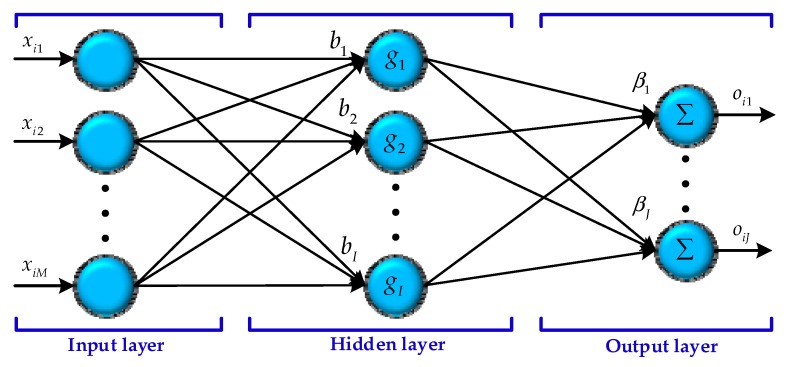
Architecture of the extreme learning machine.

An extreme learning machine is a single hidden layer feed-forward neural network (SLFNN) algorithm, the most prominent feature of which is that it can be faster than the traditional neural network algorithm under the premise of ensuring learning accuracy. Moreover, it can randomly generate the link weights between the input layer and the hidden layer as well as the thresholds of the hidden layer neurons which need not be adjusted in the training process so that the optimal solution can be obtained by only setting the number of neurons in the hidden layer [28,29]. The architecture of the ELM is illustrated in Figure 3.

There are three layers in ELM—the input layer, the hidden layer, and the output layer from Figure 3—that have M input neurons, I hidden neurons, and J output neurons. Supposing there are N samples $\left(x_{i},t_{i}\right)$, where $x_{i}={\left[x_{i1},x_{i2},\cdots ,x_{iM}\right]}^{T}\in R^{M}$ and $o_{i}={\left[o_{i1},o_{i2},\cdots ,o_{iJ}\right]}^{T}\in R^{J}$, an ELM with I hidden neurons can be expressed as

(28) $$o_{j}=\sum_{i=1}^{I}\beta_{i}g\left(w_{i}\cdot x_{j}+b_{i}\right),\;\left(j=1,2,\cdots ,N\right)$$

(29) $$g\left(x\right)=\frac{1}{1+e^{-x}}$$

where g(x) is the activation function, which is often used as a sigmoid function in traditional ELM and is also selected in this paper; $w_{i}={\left[w_{i1},w_{i2},\cdots ,w_{iM}\right]}^{T}$ denotes the weight between the input neurons and the hidden neurons; $\beta_{i}={\left[\beta_{i1},\beta_{i2},\cdots ,\beta_{iI}\right]}^{T}$ represents the weight connecting the hidden neurons and the output neurons; and $b_{i}$ is the bias of the hidden neuron. The stand SLFNN tries to minimize the difference between $o_{j}$ and $t_{j}$, which can be expressed as

(30) $$\sum_{i=1}^{I}\beta_{i}g\left(w_{i}\cdot x_{j}+b_{i}\right)=t_{j},\;\left(j=1,2,\cdots ,N\right).$$

At the same time, Equation (30) can also be expressed as a matrix:

(31) Hβ=T

where H is the output of the hidden layer neuron; β is the output weight; and T is the target matrix of the N training samples.

(32) $$H\left(w_{1},\cdots ,w_{I},b_{1},\cdots ,b_{I},x_{1},\cdots ,x_{N}\right)=\left[\begin{array}{ccc} g\left(w_{1}x_{1}+b_{1}\right) & \cdots & g\left(w_{I}x_{1}+b_{1}\right)\\ \vdots & \cdots & \vdots \\ g\left(w_{1}x_{N}+b_{1}\right) & \cdots & g\left(w_{I}x_{N}+b_{I}\right) \end{array}\right].$$

The training target is to meet the following requirements:

(33) $$\left\|H\left(w_{1},\cdots ,w_{I},b_{1},\cdots ,b_{I}\right)\hat{\beta }-T\right\|=\underset{\beta }{\min}\left\|H\left(w_{1},\cdots ,w_{I},b_{1},\cdots ,b_{I}\right)\beta -T\right\|.$$

Thus, the output weight vector can be calculated by the smallest norm least square solution as follows:

(34) $$\hat{\beta }=H^{†}T$$

where $H^{†}$ is the Moore–Penrose generalized inverse of matrix H. The specific steps of the extreme learning machine are shown in Table 2.

### 3.4. The Improved Extreme Learning Machine

One of the biggest problems for the traditional extreme learning machine is that the activation function is fixed which leads to poor convergence of network training [30]. Therefore, an improved extreme learning machine is proposed in this section, which uses a Fourier orthogonal basis function instead of a sigmoid function for network activation function.

Any nonlinear function y=f(x) can be represented linearly by a set of orthogonal basis functions:

(35) $$y=f\left(x\right)=\sum_{i=1}^{I}\omega_{i}\cdot g_{i}\left(x\right)+R\left(x\right)=W^{T}G\left(x\right)+R\left(x\right)$$

where G(x) is the orthogonal basis function; W is the correlation coefficient; and R(x) is the approximation accuracy error. According to Equation (35), the ELM mathematical model based on a Fourier orthogonal basis function can be expressed as

(36) $$\hat{y}=\hat{f}\left(x\right)=\sum_{i=1}^{I}\beta_{i}\cdot g_{i}\left(x\right)+R\left(x\right)=\beta^{T}G\left(x\right)+R\left(x\right)$$

where $G\left(x\right)=\left[g_{1}\left(x\right),g_{2}\left(x\right),\cdots ,g_{I}\left(x\right)\right]$. Substituting a Fourier orthogonal basis function for a sigmoid activation function in Formula (29) gives

(37) $$g_{i}\left(x\right)=\{\begin{array}{cc} 1 & i=0\\ \cos\left(\frac{i+1}{2}\cdot \frac{\pi x}{l}\right) & i=1,3,5,\cdots \\ \sin\left(\frac{i}{2}\cdot \frac{\pi x}{l}\right) & i=2,4,6,\cdots \end{array}$$

where i denotes ith hidden neuron. The IELM has a different activation function for each neuron, which improves the training convergence rate while ensuring the training accuracy.

## 4. System Structure for GPS and INS

**Figure 4:**
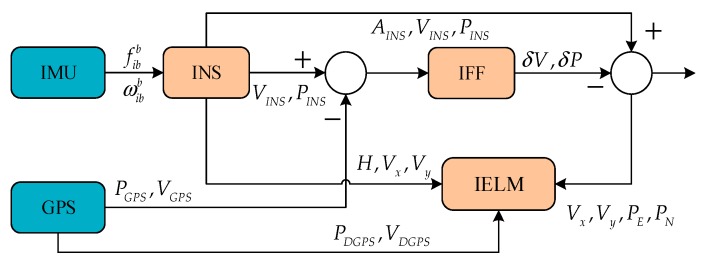
Training mode based on the improved extreme learning machine (IELM) when GPS data is available. IFF: improved fading filter.

**Figure 5:**
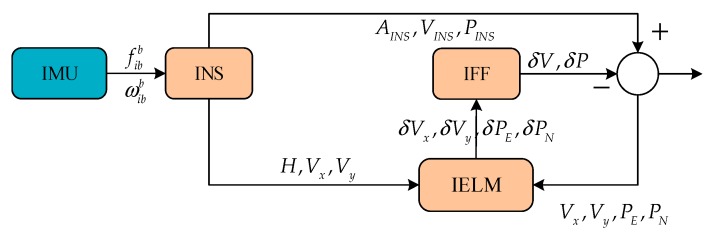
Prediction mode based on the IELM during GPS outages.

In the proposed system structure for GPS and INS, the IELM works in two modes. The IELM works in training mode when GPS signals are available, as shown in Figure 4. Through the specific force $f_{ib}^{b}$ and angular velocity $\omega_{ib}^{b}$ measured from IMU, the attitude $A_{INS}$, speed $V_{INS}$, and position $P_{INS}$ information of the vehicle’s motion are calculated by the INS. Meanwhile, the heading H, speed $V_{x}$, and speed $V_{y}$ are selected as the inputs of the IELM. In this system, the GPS provides the pseudo-range position and speed, which are used to loosely couple the navigation with the INS information, while the pseudo-GPS position and pseudo-GPS velocity information are collected as the inputs of the IELM. The proposed IFF algorithm is used to process the speed errors and position errors between INS and GPS. Finally, $V_{x},V_{y},P_{E}$ and $P_{N}$ are used in the final output of the system as the inputs of the IELM. The velocity error and the position error are introduced into the IELM as the target vectors of the training. Another mode is the prediction mode, which is based on the IELM in the presence of GPS outage, as shown in Figure 5. $\delta V_{x},\delta V_{y},\delta P_{E}$, and $\delta P_{N}$ are predicted by IELM as input information of the IFF algorithm. We can obtain the final output through the INS and the IFF-processed δV,δP during GPS signals outages.

## 5. Discussion

### 5.1. Simulation Test

The proposed algorithm was simulated under the GPS/INS loosely coupled mode. The biases caused by drift and the random walk noise of the accelerometer were set as 100 μg and $100\;\mu g/\sqrt{Hz}$, respectively. The biases and random walk noise of the gyroscope were set as $0.02^{{}^{\circ}}/h$ and $0.02^{{}^{\circ}}/\sqrt{h}$, respectively. The initial misalignment angle was set as $0.01^{{}^{\circ}}$ for heading, pitching, and roll. The GPS speed and position errors were set as 1 m and 0.1 m/s, respectively. The out frequency of the inertial sensors and GPS receiver were set as 100 Hz and 1 Hz, respectively. The vehicle movement start position was set to latitude $32.05^{{}^{\circ}}N$ and longitude $118.79^{{}^{\circ}}E$. The process of the vehicle’s motion is listed in Table 3.

**Figure 6:**
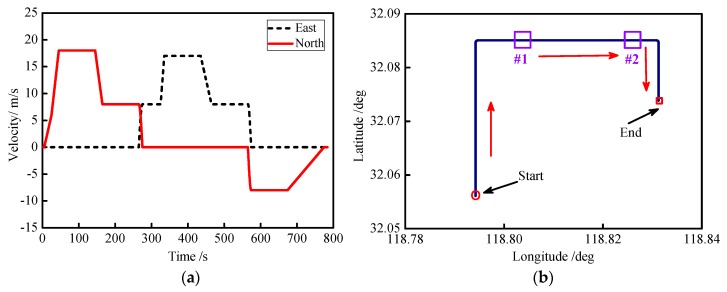
Curves of vehicle motion: (**a**) east and north vehicle velocities; (**b**) curves of the moving trajectories.

The moving speeds of the vehicle and trajectories are shown in Figure 6a,b; the velocities of the vehicle in the east and north directions were less than 20 m/s, respectively. The whole trajectory had two turns representing rotating speeds of 9°/s and 10°/s, respectively. In order to verify the performance of GPS/INS integrated positioning when GPS was out of lock, two simulated GPS outages are marked by purple lines in Figure 6b, which represent GPS outages times of 50 s (350~400 s) and 100 s (450~550 s).

**Figure 7:**
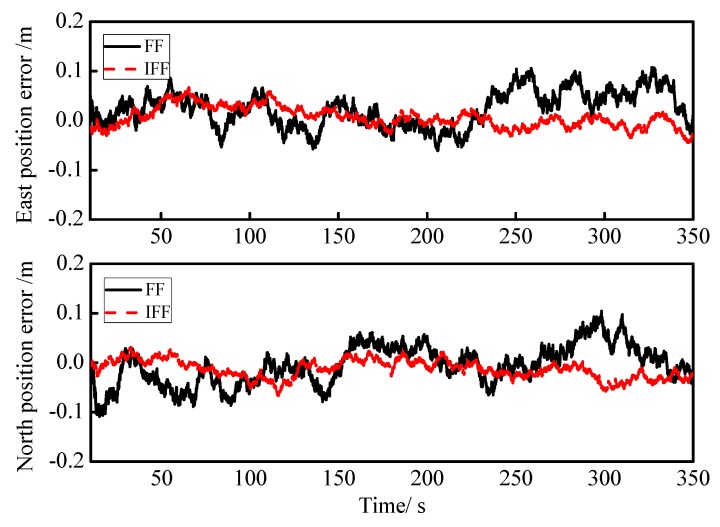
Curves of the east and north position errors for the fading filter (FF) and the IFF.

First, the IFF algorithm proposed in this paper was verified. In the IFF algorithm, the select step size was set as u=0.01, error variance ratio threshold was m=1.3, and the minimum value of fading factor was $S_{\min}=0.6$. In the conventional fading filter (FF), the fading factor was selected to be S=0.9. The curves of the east and north position errors for the fading filter (FF) and IFF algorithms are shown in Figure 7, in which the FF and IFF are marked by black and red lines, respectively, and the simulation time is 0s to 350s. In Figure 7, we can intuitively see that the red line is less volatile than the black line, so the IFF performed better than the FF. To compare the performance of each algorithm in a clearer way, the root-mean-square errors (RMSEs) of the east and north positions for each algorithm were calculated, and they are listed in Table 4, showing that the RMSE position of the IFF algorithm was about half the RMSE position of the FF algorithm.

**Figure 8:**
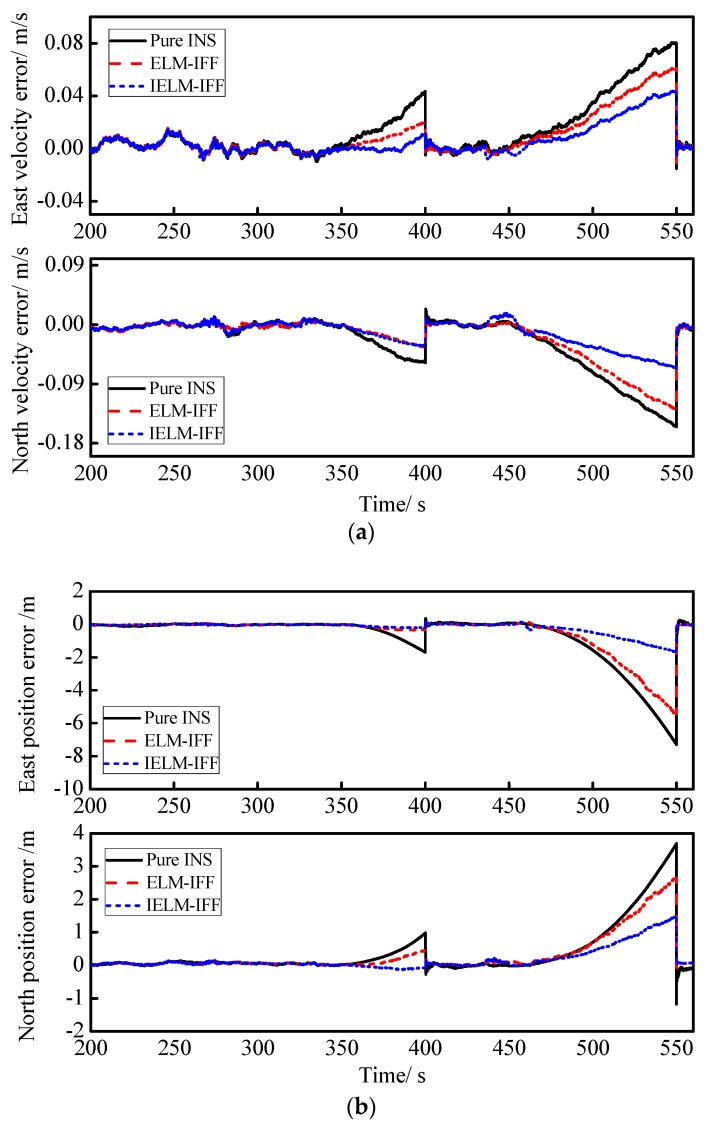
Curves of velocity and position errors: (**a**) east and north velocity errors for pure INS, ELM-IFF, and IELM-IFF; (**b**) east and north position errors for pure INS, ELM-IFF, and IELM-IFF. ELM: extreme learning machine.

In order to verify the validity of the IELM+IFF algorithm proposed in this paper during GPS outages, two GPS outages were simulated for (#1) 350s~400 s and (#2) 450s~550 s. Figure 8a shows the east and north velocity errors for pure INS, ELM-IFF, and IELM-IFF. Meanwhile, Figure 8b displays the east and north position errors for pure INS, ELM+IFF, and IELM-IFF. Figure 8a,b clearly demonstrate that when the IFF algorithm is used at the same time, the improved ELM algorithm for the suppression of speed errors and position errors is obviously superior to the traditional ELM algorithm during GPS outages.

The RMSEs of the velocities and positions from the pure INS, ELM-IFF, and IELM-IFF with the first and second GPS outages periods are listed in Table 5. The result show that the proposed IELM-IFF algorithm was more effective than ELM-IFF as it decreased the RMSEs of the east and north velocities by about 38% and 60%, while the RMSEs of the east and north positions decreased by about 45% and 43%, respectively.

### 5.2. Real Road Test

To evaluate the performance of the proposed algorithm compared to the conventional counterparts in practical applications, a real road test was designed and is detailed in this subsection. The vehicle test equipment included an inertial measurement unit (IMU), a GPS receiver, PHINS, and a computer. The IMU consisted of three fiber optic gyroscopes and three accelerometers; the GPS receiver used the FlexPark6 GPS receiver, PHINS; from the French IXBLUE Inertial Navigation system; and the computer used was PC104. PHINS was used to provide accurate navigation reference information. The detailed performance parameters of IMU and GPS receiver are shown in Table 6, and the PHINS specifications are listed in Table 7.

**Figure 9:**
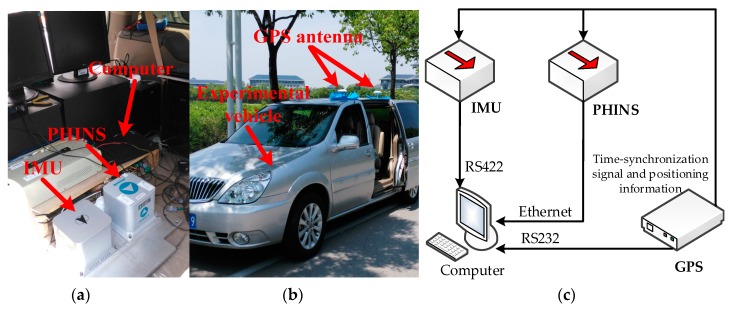
(**a**) PHINS and IMU installation diagram; (**b**) experimental vehicle; (**c**) structure of the vehicle test equipment.

As Figure 9a shows, the PHINS and IMU were installed together, Figure 9b shows the experimental vehicle, and Figure 9c shows the structure of the vehicle test equipment. We can see from Figure 9b that the outputs of GPS provided the time-synchronization signal for PHINS and IMU. The raw data of the outputs of the IMU were transferred via an RS422 port, and the PHINS data were collected via Ethernet from the computer. The GPS data was acquired through an RS232 communication interface. At the same time, a real-time operation system, VxWorks, was embedded in the computer.

**Figure 10:**
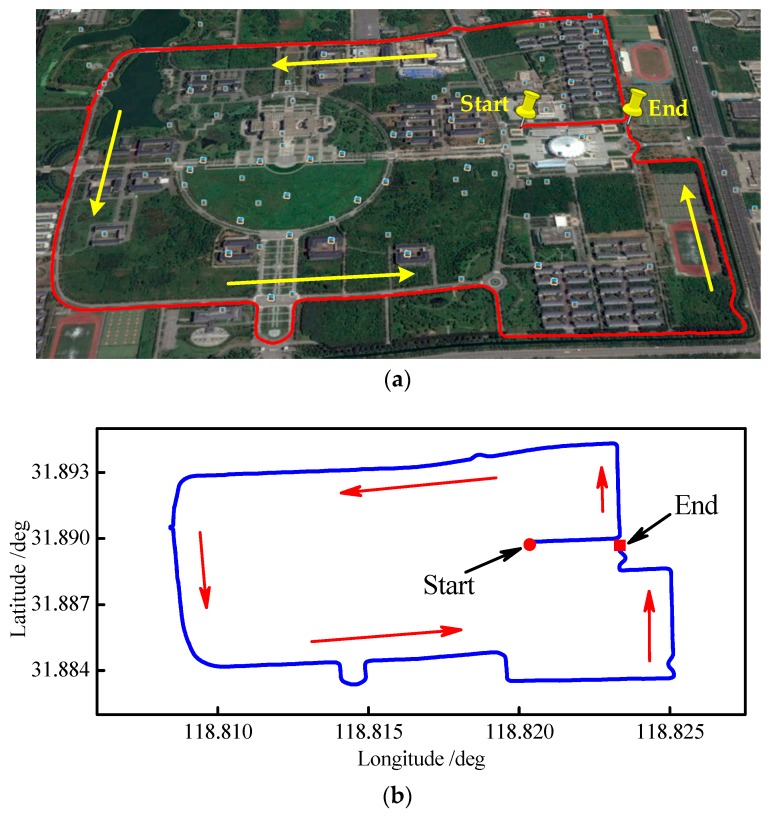
Vehicle trajectory. (**a**) Google map of the reference trajectory; (**b**) coordinates of the reference trajectory.

Figure 10 shows the vehicle trajectory, which was tested at the Jiulonghu campus of Southeast University in Nanjing. Figure 10a shows the Google map of the reference trajectory. Meanwhile, Figure 10b shows the coordinates of the reference trajectory.

**Figure 11:**
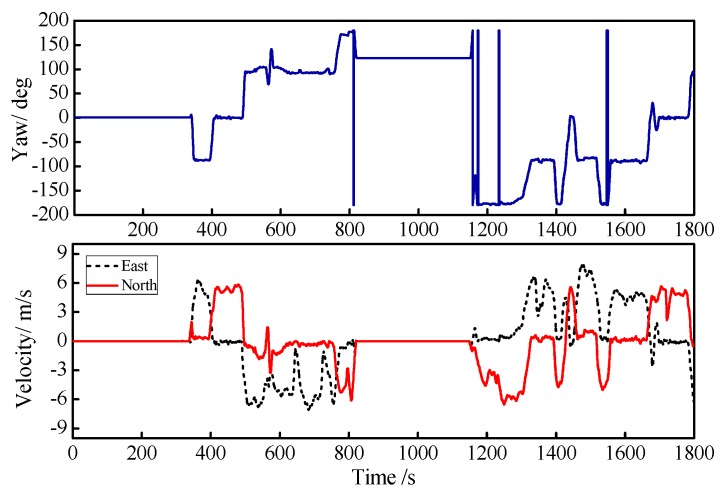
Curves of the yaw, east velocity, and north velocity.

The initial alignment time of the system was 0~300 s; after 300s, the whole system worked under the GPS/INS loosely coupled mode. The entire testing process took 1850 s and the GPS signal was good under the test environment. The yaw angle, east velocity, and north velocity information for the entire exercise are shown in Figure 11, where the yaw angle is depicted by the blue line, and the east velocity and the north velocity are described by the black and red lines, respectively.

**Figure 12:**
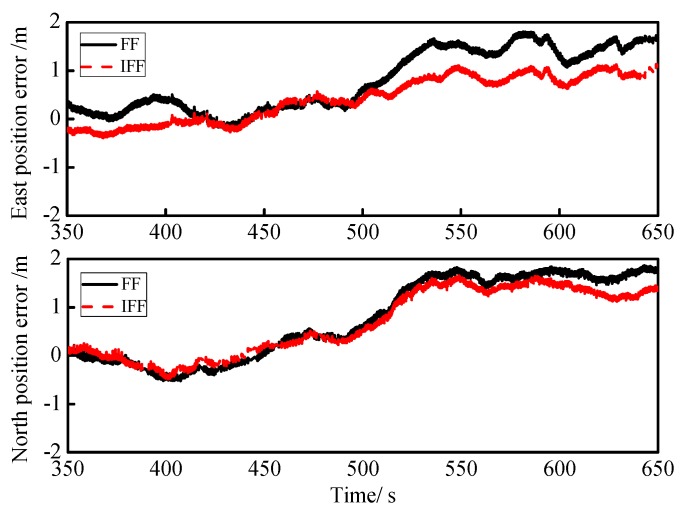
Curves of the east and north position errors for FF and IFF.

The proposed algorithm was verified by the data collected in the above real road test. First, the IFF algorithm and FF algorithm were verified by the east position error and the north position error of GPS/INS integrated navigation from 350 s to 650 s, which is shown in Figure 12. In the IFF algorithm, the step size was u=0.005, the error variance ratio threshold was m=1.3, and the minimum value of the fading factor was $S_{\min}=0.6$, while in FF, the fading factor was S=0.9. In order to more accurately illustrate the superiority of the IFF algorithm, the RMSEs of the east and north positions were obtained by calculating the root-mean-square error of the error data in Figure 12, which are listed in Table 8. Compared with the FF algorithm, the RMSEs of the east position and north position of the IFF algorithm (0.6319 and 0.9639) reduced by about 38% and 15%, respectively.

**Figure 13:**
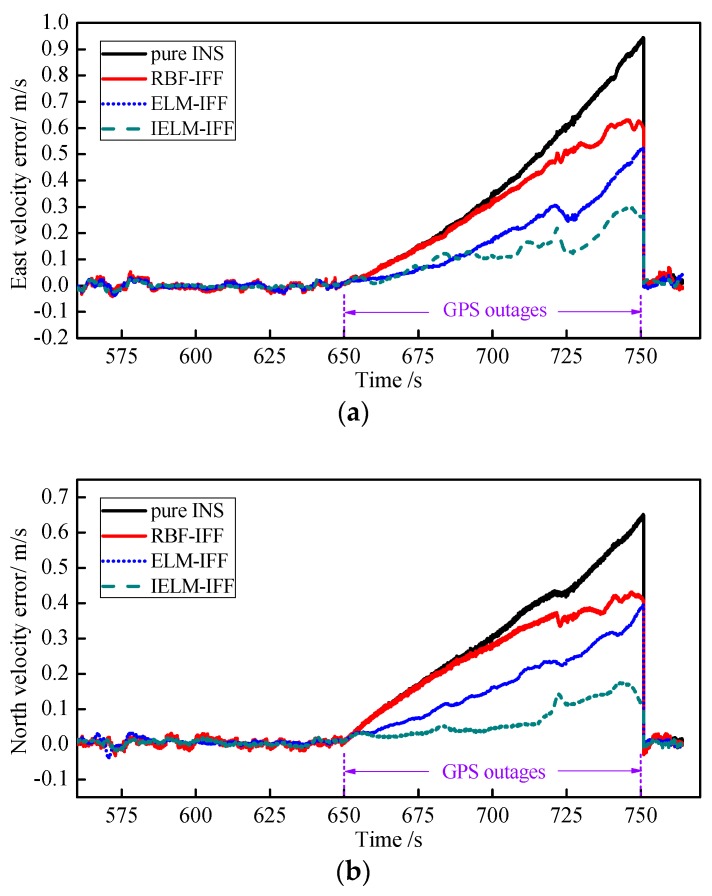
Curves of the east velocity error (**a**) and north velocity error (**b**) during GPS outages.

**Figure 14:**
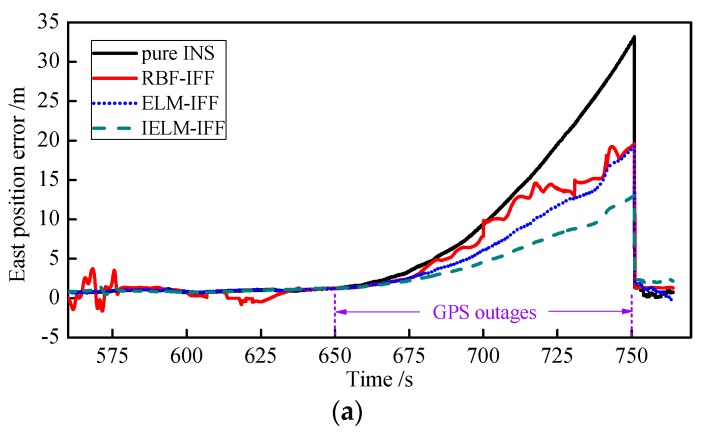

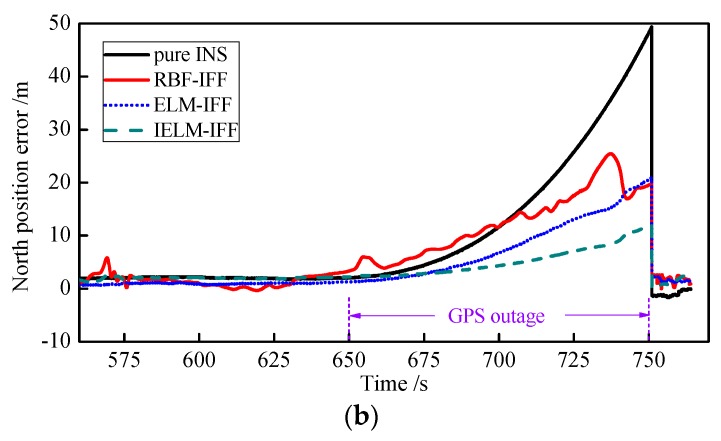
Curves of the east position error (**a**) and north position error (**b**) during GPS outages.

Second, to validate the performance of the IELM algorithm, the results were compared with the ELM and RBF neural network algorithms. The number of hidden layer nodes in the IELM, ELM, and RBF neural network was all 20. The training time was 300 s to 650 s, and the simulation set the GPS outage time from 650 to 750 s. The east velocity error and north velocity error for the pure INS, RBF-IFF, ELM-IFF, and IELM-IFF of GPS outages are shown in Figure 13a,b. The error gradually increased from 650 s to 750 s in the order of pure INS, RBF-IFF, ELM-IFF, and then IELM-IFF during the GPS outages. The east position error and north position error for the pure INS, RBF-IFF, ELM-IFF, and IELM-IFF during the GPS outages are shown in Figure 14a,b. Compared with other algorithms, the IELM-IFF algorithm had the smallest position error when the GPS signal lost lock. To compare the performance of each algorithm in a clearer way, the root-mean-square error and maximum error of the velocity and position information for each algorithm during the GPS outages are listed in Table 9. The maximum errors and RMSEs of the velocity and position for IELM-IFF were the smallest compared with the other algorithms.

**Figure 15:**
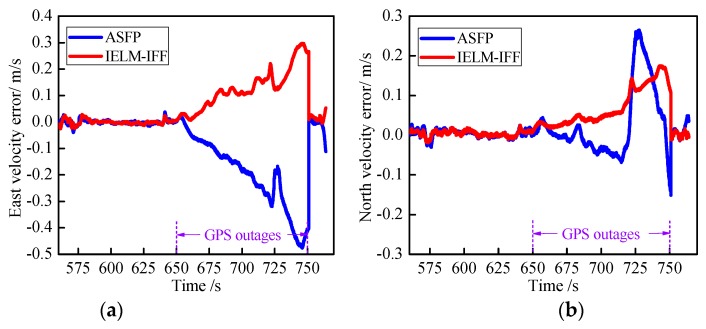

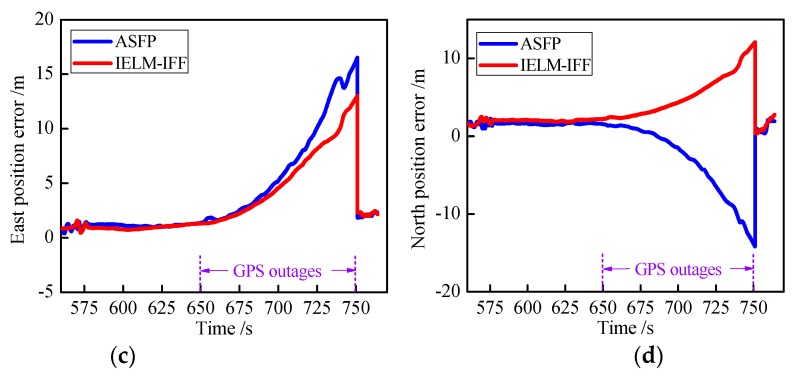
Curves of the east velocity error (**a**), north velocity error (**b**), east position error (**c**), and north position error (**d**) during GPS outages.

Another existing algorithm was compared to demonstrate the effectiveness of the proposed approach. The artificial-intelligence-based segmented forward predictor (ASFP) was developed in reference [31] and uses two RBFNNs and a forward prediction algorithm. The ASFP algorithm was compared with the proposed IELM-IFF algorithm. Figure 15 presents the results during GPS outages from 650 to 750 s, where Figure 15a,b show the east and north velocity errors, respectively, and Figure 15c,d show the east and north position errors, respectively. The maximum errors of the east and north positions were 13.0378 and 12.0901 for the IELM-IFF algorithm. However, the maximum position errors for the ASFP algorithm were 16.5424 and –14.1796. So, the proposed IELM-IFF showed better precision than the ASFP algorithm, and the east and north position errors reduced by 21.18% and 14.73%, respectively.

**Figure 16:**
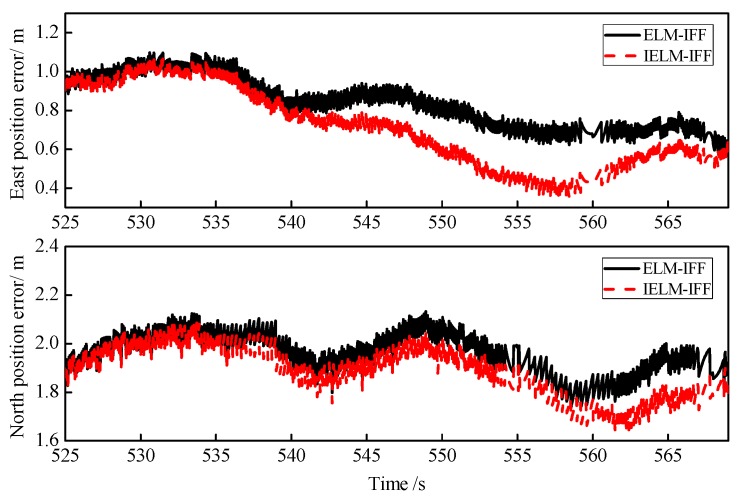
Curves of position errors for ELM-IFF and IELM-IFF in the case of good GPS signal.

Third, Figure 16 shows the position prediction errors for ELM-IFF and IELM-IFF in the case of good GPS signal with training times from 300 to 525 s and a forecast period of 525 to 570 s. In addition, the RMSEs of the position for these two algorithms under good GPS signal are listed in Table 10. The RMSEs of the east position and north position predictions were 0.7346 and 1.8919, which is better than the results for the ELM-IFF algorithm.

**Figure 17:**
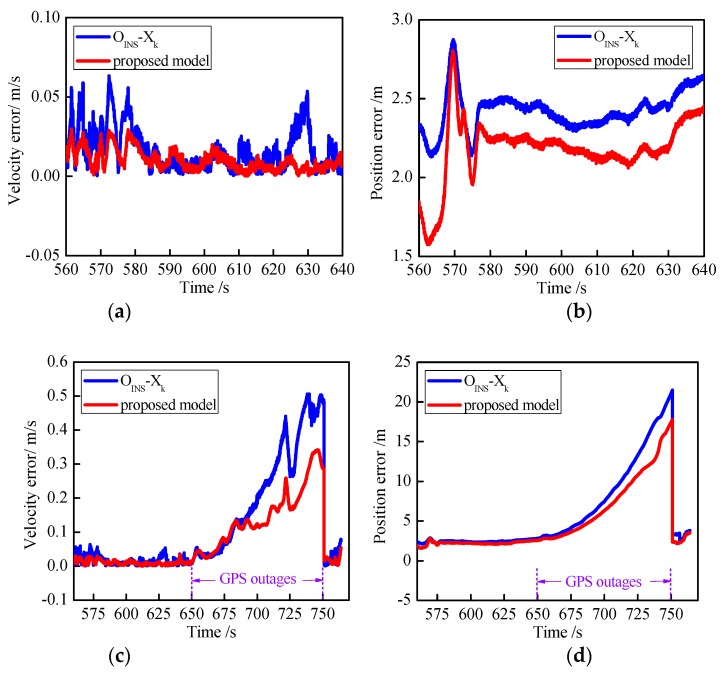
Curves of the velocity and position errors: velocity error (**a**) and position error (**b**) with a good GPS signal; velocity error (**c**) and position error (**d**) during GPS outages.

Fourth, to evaluate the performance of the proposed model, the $O_{INS}-X_{k}$ model was utilized as a comparison [32]. Figure 17 displays the horizontal velocity error and the horizontal position error, where Figure 17a,b represent good GPS signals, and Figure 17c,d represent GPS outages from 650 to 750 s. When the GPS signal was good, the proposed model showed better accuracy than the $O_{INS}-X_{k}$ model in most cases. When the GPS signal was unavailable for 650 to 750 s, the two models showed different results. It is obvious that the proposed model outperformed the $O_{INS}-X_{k}$ model. During the GPS outages for a period of 650 to 680 s, the velocity errors of the $O_{INS}-X_{k}$ model and proposed model were similar. This means that during short GPS outages, both models could be employed to reduce the velocity error. However, when the GPS outage becomes longer, the proposed model achieves higher accuracy than the $O_{INS}-X_{k}$ model.

The reason why the proposed model performs better than the $O_{INS}-X_{k}$ model is that the predicted pseudo GPS position only relates to the output of INS, while the predicted state vector $X_{k}$ is influenced by both the INS information and the accuracy of the loosely-coupled KF. When the last estimation of the KF is correct, the $O_{INS}-X_{k}$ can be well utilized. However, the GPS/INS integrated system cannot ensure absolute accuracy, which means there are always small errors in the estimation of the KF.

Fifth, in order to compare the computational costs between different algorithms, RBF, ELM-IFF, ASFP, and IELM-IFF with the same number of nodes were investigated. To facilitate the observation of the computational cost, the number of pieces of training sample data was set as 100. The average time consumption of training procedures for each algorithm in the simulation is listed in Table 11, showing that the ELM-IFF, ASFP, and IELM-IFF performed faster than RBF. The computational cost of the proposed IELM-IFF algorithm was 4.71ms, which is almost similar to the ELM-IFF algorithm. So, the IELM-IFF algorithm performed better than ELM-IFF and, at the same time, it did not increase the computational cost. In addition, the IELM-IFF method was shown to have a lower computational coast than the ASFP algorithm proposed in reference [31].

**Figure 18:**
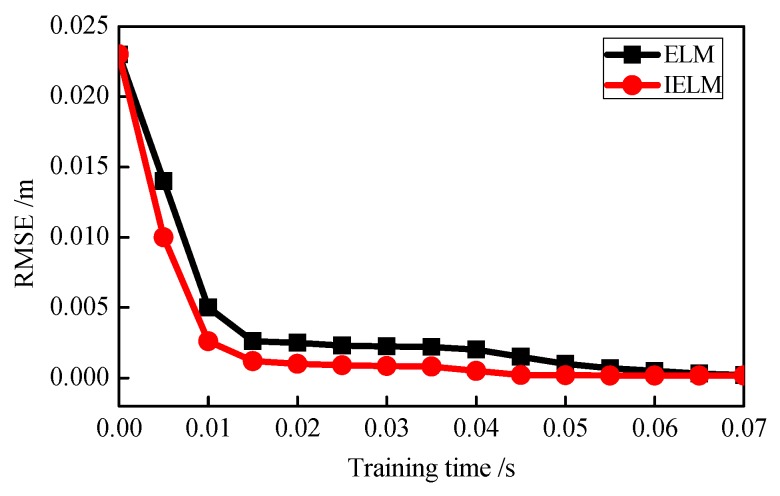
Curves of convergence performance.

Finally, Figure 18 shows the curves of convergence performance for the ELM and IELM algorithms, in which the ordinate represents the RMSE values during training, and the abscissa indicates the training times. It can be seen from Figure 16 that the IELM algorithm achieved higher convergence accuracy than the ELM algorithm for the same training time.

## 6. Conclusions

In this paper, a new algorithm was proposed for GPS/INS integrated navigation during GPS outages based on a fading filter and an extreme learning machine, and a new training model strategy was developed. An improved fading filter algorithm was also proposed with the aim of adjusting the fading factor in the traditional forgetting filter. This algorithm can dynamically adjust the fading factor so that the fading filter can achieve a better filtering effect in real-time. In order to solve the problem of the rapid divergence of a GPS/INS loosely coupled navigation system during GPS outages, this paper introduced the ELM algorithm into the integrated navigation system, which greatly improved the speed of the network training compared with the traditional radical basis function (RBF) neural network. In order to solve the problems of the fixed activation function in the ELM algorithm and the slow convergence speed, this paper presented an improved ELM algorithm based on a Fourier orthogonal basis function.

In order to verify the performance of the proposed algorithm, this paper proposed simulation experiments and a real road vehicle test. Regarding the IFF algorithm verification, by comparing and analyzing the FF algorithm under good GPS signal conditions, the IFF algorithm was shown to have a better filtering effect. The performance of the IELM algorithm was verified by the training time, prediction accuracy, and convergence speed, and compared with a traditional RBF neural network. The results showed that compared with the RBF and ELM algorithms, IELM can reduce the divergence of inertial navigation errors and achieve higher positioning accuracy.

## Figures and Tables

**Table 1:** The state vector with 15 dimensions.

State	Definition	Coordinate System
$\phi_{E},\phi_{N},\phi_{U}$	Attitude errors (misalignment angles)	Geographical frame g
$\delta V_{E},\delta V_{N},\delta V_{U}$	Velocity errors	Geographical frame g
δL,δλ,δh	Position errors	Geographical frame g
$\nabla_{x},\nabla_{y},\nabla_{z}$	Accelerometer biases	Body Frame b
$\varepsilon_{x},\varepsilon_{y},\varepsilon_{z}$	Gyro biases	Body Frame b

**Table 2:** Extreme learning machine algorithm.

Number	Content
Step 1	Giving the training samples set $\left(x_{i},t_{i}\right)$, activation function g(x), and hidden neurons I
Step 2	Randomly generate hidden layer node parameters $\left(w_{i},b_{i}\right)$
Step 3	Calculate the hidden layer output matrix H
Step 4	Calculate the output weight vector $\beta =H^{†}T$

**Table 3:** The process of the vehicle’s motion.

Time (s)	State	Time (s)	State
0–5	Stationary state	325–335	Accelerated motion (a = 0.9 m/s^2^)
5–25	Accelerated motion (a = 0.3 m/s^2^)	335–435	Uniform motion (v = 17 m/s)
25–45	Accelerated motion (a = 0.6 m/s^2^)	435–465	Decelerated motion (a = −0.3 m/s^2^)
45–145	Uniform motion (v = 18 m/s)	465–565	Uniform motion (v = 8 m/s)
145–165	Decelerated motion (a = −0.5 m/s^2^)	565–574	Turn right motion (w = 10°/s)
165–265	Uniform motion (v = 8 m/s)	574–674	Uniform motion (v = 8 m/s)
265–275	Turn right motion (w = 9°/s)	674–774	Decelerated motion (a = −0.08 m/s^2^)
275–325	Uniform motion (v = 8 m/s)	774–784	Stationary state

**Table 4:** Position (m) root-mean-square error (RMSE) for the FF and the IFF.

Method	East Position	North Position
FF	0.0429	0.0404
IFF	0.0219	0.0226

**Table 5:** The RMSEs of the velocity (m/s) and position (m) from the pure INS, ELM-IFF, and IELM-IFF.

GPS Outage	Method	East Velocity	North Velocity	East Position	North Position
#1	Pure INS	0.0224	0.0381	0.0451	3.1604
	ELM-IFF	0.0104	0.0230	0.0341	2.4498
	IELM-IFF	0.0035	0.0210	0.0235	0.8027
#2	Pure INS	0.7808	0.4320	0.0871	1.5541
	ELM-IFF	0.2461	0.2006	0.0719	1.2410
	IELM-IFF	0.1526	0.0779	0.0384	0.7068

**Table 6:** Performance of the inertial measurement unit (IMU) and the GPS receiver.

Sensors	Rand Biases	Rand Walk	Data Rate	RMSE
Gyro	$\leq 0.02^{{}^{\circ}}/h$	$\leq 0.02^{{}^{\circ}}/\sqrt{h}$	200 Hz	-
Accelerometer	≤6 mg	$\leq 100\;\mu g/\sqrt{Hz}$	200 Hz	-
GPS receiver	-	-	1 Hz	Position: 2 m; Velocity: 0.1 m/s

**Table 7:** PHINS specifications.

Performance of PHINS	
No aid for 2 min/5 min	3 m/20 m
Pure inertial mode	0.6 nm/h
With GPS/Ultra Short Base Line (USBL)/Long Base Line (LBL)	0.01° secant latitude
Roll and pitch dynamic accuracy (no aid)	0.01°
Data output rate	200 Hz

**Table 8:** RMSEs of the positions (m) for FF and IFF.

Method	East Position	North Position
FF	1.0202	1.1339
IFF	0.6319	0.9639

**Table 9:** The RMSEs and maximum error values of the velocity (m/s) and position (m) for the different algorithms. ASFP: artificial-intelligence-based segmented forward predictor.

		Pure INS	RBF-IFF	ELM-IFF	ASFP	IELM-IFF
East velocity	Max-Error	0.9442	0.6310	0.5261	–0.4763	0.2967
	RMSE	0.3348	0.2653	0.1673	0.1689	0.1036
North velocity	Max-Error	0.6515	0.4315	0.4148	0.2650	0.1749
	RMSE	0.2510	0.2025	0.1709	0.0633	0.0600
East position	Max-Error	49.3993	25.4681	20.9953	16.5424	13.0378
	RMSE	15.1727	9.8322	7.1433	4.2778	4.5788
North position	Max-Error	33.2024	19.5778	19.2637	–14.1796	12.0901
	RMSE	10.9098	7.5333	6.4667	5.9954	4.3585

**Table 10:** Position (m) RMSE for different algorithms in the case of good GPS signal.

Method	East Position	North Position
ELM-IFF	0.8456	1.9618
IELM-IFF	0.7346	1.8919

**Table 11:** Time consumption of different algorithms.

Method	Time Consumption (ms)
RBF	6.72
ELM-IFF	4.69
ASFP	5.05
IELM-IFF	4.71
